# Clinical Study of Intradermal Injection of Non‐Crosslinked Sodium Hyaluronate Combined With Human Epidermal Growth Factor in the Treatment of Skin Barrier Injury in Plateau Area

**DOI:** 10.1111/jocd.16727

**Published:** 2024-12-24

**Authors:** Ao He, Boyan Liu, Yingjian Hua, Zhuo Gong, Fengshan Gan, Qingzhu Zhou, Songmei Wang, Xian Zhao

**Affiliations:** ^1^ Department of Plastic Surgery Affiliated Calmette Hospital of Kunming Medical University Kunming China; ^2^ Department of Medical Cosmetology Kunming University of Science and Technology Affiliated Pu'er Hospital Pu'er Yunnan China; ^3^ School of Public Health Kunming Medical University Kunming Yunnan China

**Keywords:** actinic damage, aesthetic medicine, epidermal growth factors, hyaluronic acid, plastic surgery, UV damage

## Abstract

**Background:**

The Yunnan–Guizhou Plateau's high‐altitude setting is characterized by intense solar ultraviolet radiation, a significant environmental stressor that frequently leads to skin barrier damage. This damage presents clinically as erythema, itching, and desquamation, underscoring the need for effective reparative interventions.

**Aims:**

The objective of this study was to assess the therapeutic efficacy of a novel treatment protocol that integrates non‐crosslinked hyaluronic acid (HA) injection with microneedle application of human epidermal growth factor (hEGF) for the restoration of skin barrier function in regions of high altitude.

**Methods:**

Sixty female subjects exhibiting characteristic signs of skin barrier impairment were randomized into four cohorts: a control group and three experimental groups differentiated by hEGF concentration. The intervention comprised subdermal HA injection coupled with microneedle therapy. The VISIA digital skin analysis system was utilized to quantify skin barrier integrity, with assessments performed by a panel of dermatologists and through patient self‐evaluations at baseline and postintervention.

**Results:**

A marked reduction in erythema and indices of skin barrier damage was observed in the experimental groups relative to the control. The cohort administered 10 000 IU hEGF exhibited the most pronounced restoration of skin barrier function, indicative of a dose‐dependent therapeutic response. The treatment demonstrated favorable tolerability without any reported adverse events.

**Conclusions:**

The conjoint application of non‐crosslinked HA and hEGF presents as a potent therapeutic modality for the repair of the skin barrier in high‐altitude environments. Our findings indicate that an optimized concentration of hEGF is pivotal for achieving the most efficacious treatment outcomes.

## 1 Introduction

Yunnan Province, located on the Yunnan–Guizhou Plateau, is distinguished by its high altitude and thin air, which contribute to significant solar penetration and intense ultraviolet (UV) radiation, especially during the winter and spring [1]. This environmental condition is a prevalent cause of skin barrier damage among the local populace. The skin, the body's largest organ, serves a crucial barrier function, underpinned by the “brick and mortar” model comprising keratinocytes, intercellular lipids, and the sebum film [2]. The stratum corneum, as the foremost defensive layer, is pivotal in preventing skin dryness, offering mechanical protection, and safeguarding the underlying cells from UV‐induced damage, thereby modulating inflammatory responses and preserving skin hydration [3, 4, 5]. However, exposure to high‐intensity UV radiation can impair the dermal–epidermal junction, deplete ceramides, and attenuate the stratum corneum's thickness, culminating in a weakened skin barrier and accelerated extrinsic skin aging [6]. Clinical manifestations include facial erythema, pruritus, desquamation, and a propensity for inflammatory skin conditions [7, 8]. Current strategies to counteract photoaging encompass a range of nonsurgical treatments, such as topical medications and photodynamic therapies, alongside surgical options [9]. Human epidermal growth factor (hEGF) is recognized for its role in stimulating epidermal growth and keratinization, thereby enhancing keratinocyte proliferation and repair, accelerating re‐epithelialization, and aiding in the restoration of the epidermal structure. Hyaluronic acid (HA) is renowned for its hydrating properties in addition to its capacity to neutralize reactive oxygen species, inhibit lipid peroxidation, and exert anti‐inflammatory and antiapoptotic effects, which are instrumental in restoring the skin's barrier function. Expanding on the foundational work of Yun Seop Kim [10], our study introduces a novel therapeutic approach that integrates non‐crosslinked HA injection with microneedle application of hEGF. This strategy is designed to foster keratinocyte proliferation and repair while creating an optimal hydrated microenvironment conducive to cell growth and migration [11]. Utilizing a comprehensive set of subjective and objective assessment tools, our study endeavors to present a more efficacious and expedited treatment regimen aimed at skin barrier restoration for the high‐altitude Yunnan population.

## 2 Materials and Methods

### 2.1 Clinical Data

This study recruited a sample of 60 female subjects with typical symptoms of skin barrier damage, such as erythema, itching, telangiectasia, pigmentation, and enlarged pores, from the outpatient department of the affiliated Calmette Hospital of Kunming Medical University between January 2022 and July 2022. All subjects met the inclusion and exclusion criteria for the study (Table 1). We hereby confirm that the ethical policies of the journal, as indicated on the journal's author guidelines page, have been strictly adhered to and the requisite approval from the appropriate ethical review committee has been obtained. The research was sanctioned by the Ethics Committee of the affiliated Calmette Hospital of Kunming Medical University, and all participants furnished informed consent.

**TABLE 1:** Inclusion and exclusion criteria for this study.

Criteria	Inclusion	Exclusion
Geographical	Long‐term residence in the Yunnan Plateau region	—
Symptomatic	Skin symptoms of barrier damage: itching, redness, telangiectasia, dryness, and desquamation	—
Diagnostic	VISIA red region feature count ≥ 50	—
Treatment	No other skin treatments in the past 6 months or during the study: Botox, laser, oral isotretinoin, hyaluronic acid (HA) fillers, or mesotherapy	—
Pregnancy/lactation	—	Pregnancy or breastfeeding
Psychological	—	Severe mental disorders or psychological diseases
Allergies	—	Allergy to HA or human epidermal growth factor (hEGF)
Medical conditions	—	Cancer, diabetes, hypertension, or other severe systemic diseases
Skin conditions	—	Local skin infection, inflammation, or other skin diseases
Compliance	—	Inability to complete the treatment as scheduled
Coagulation	—	Coagulation disorders

*Note:* According to the above inclusion and exclusion criteria, 60 female subjects with skin barrier impairment in Yunnan Province, China, were included.

### 2.2 Methods

#### 2.2.1 Medications

The study utilized medical HA gel, branded as Shuweike, with a concentration of 14 mg/mL per 2 mL vial, produced by Shanghai Qisheng Bio‐Preparation Co. Ltd., and approved with the number: Guoqi Zhun 20153140476. Additionally, topical hEGF was used, with each vial containing 20 000 IU EGF dissolved in 4 mL of physiological saline, manufactured by Shanghai Haohai Bio‐Technology Co. Ltd., and bearing the approval number S20010094. All steps in the preparation and quantification of hEGF comply with the standards set forth in the 2015 edition of the Chinese Pharmacopeia. Specifically: Production and testing facilities, raw materials, excipients, water, equipment, and animals meet the requirements outlined in the “General Rules” section of the Chinese Pharmacopeia. Each batch of hEGF underwent rigorous quality control testing, including biological activity, protein content, purity, and sterility checks, to ensure compliance with pharmacopeial standards.

#### 2.2.2 Study Methodology

Subjects were randomly allocated into four groups following the established inclusion and exclusion criteria: One control group consisting of 15 individuals and three experimental groups, each comprising 15 individuals. The control group was administered 2 mL of HA plus 1 mL of normal saline (NS). Experimental Group I received 2‐mL HA with an addition of 1 mL containing 2500 IU of hEGF. Experimental Group II was given 2‐mL HA plus 1 mL with 5000 IU hEGF, while Experimental Group III received 2‐mL HA with 1 mL of 10 000 IU hEGF. Subdermal injection of non‐crosslinked HA was performed using the Demartha first‐generation electronic injection instrument, with a maximum injection depth of 0.5 mm to prevent postinjection bleeding, considering the compromised stratum corneum of the subjects. Postinjection, full‐face microneedling was conducted using a 0.5‐mm single‐use sterile skin roller. This was followed by the application of hEGF in varying concentrations along with sterile medical hydrating facial masks, tailored to the group assignments. The treatment was administered once every 28 ± 7 days for a duration of 4 months.

The treatment procedure was standardized as follows: Prior to each session, subjects underwent comprehensive facial cleansing. Topical anesthetic cream was applied 30–40 min pretreatment and subsequently removed. In a supine position, following facial disinfection with iodine, HA gel was injected into the dermis using an electronic injector set at a slow injection speed, with a dosage of 0.025 mL per puncture, 10% negative pressure attraction, and 80% retraction force. Microneedling with the skin roller was then conducted, succeeded by the application of hEGF, and hydrating facial masks for a duration of 15–20 min. Participants were advised to avoid invasive skin aesthetic procedures (such as Botox injection, laser treatments, HA fillers, or mesotherapy) for 60 days prior to and during the trial, but were permitted to continue their usual skincare routines, including the use of noninvasive skincare products such as moisturizers, sunscreens, and mild cleansers. Strict sun protection measures were mandatory. Standardized facial observations and photographic documentation were carried out before each treatment across five facial regions: front view and both the left and right at 45 and 90 angles. VISIA digital skin analysis was also performed in a dedicated skin testing room. The conclusive assessment was executed 28 ± 7 days after the third treatment session.

#### 2.2.3 Assessment

Before each treatment session, a comprehensive skin analysis was conducted using the VISIA digital skin analysis system, with a focus on the red region feature count, a key indicator of skin barrier damage. The assessment aimed to quantify changes in this parameter as a result of the treatment. Two dermatology specialists, blinded to the treatment allocation, independently scored the subjects' photographs and VISIA skin barrier feature indicators pre‐ and posttreatment. Severity of skin barrier damage was indicated by higher scores. Concurrently, patients self‐assessed their symptoms associated with skin barrier damage, including itching, tightness, stinging, burning, flaking, and dryness, on a scale where higher scores denoted increased symptom severity. The subjective and objective evaluation data for all subjects are detailed in Tables S1–S5.

#### 2.2.4 Statistical Analysis

Statistical analysis of the collected data was executed using SPSS software version 27.0. The differences in outcomes between the treatment groups before and after the intervention were evaluated using one‐way analysis of variance (ANOVA). To ascertain the relationship between treatment frequency and skin barrier repair, repeated measures ANOVA was utilized for within‐group data analysis, thereby assessing the efficacy of the treatment over time. Statistical significance was defined as a *p*‐value of less than 0.05.

## 3 Results

### 3.1 Erythema Feature Analysis

**FIGURE 1:**
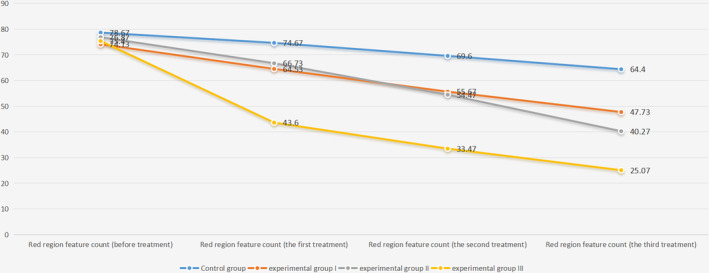
VISIA red region feature count trend plot.

The baseline comparison of erythema features among the four groups, as assessed by one‐way ANOVA of VISIA data (Table 2a; Figure 1; Figure S1), showed no statistically significant differences. Posttreatment, significant intergroup differences emerged following the first, second, and third treatments. Post hoc analysis using the least significant difference (LSD) test demonstrated a marked reduction in erythema for all experimental groups relative to the control group, with the most pronounced effect in the third group. It is important to note that no significant differences between Group I and Group II were observed until the conclusion of the third treatment (Table 2b).

**TABLE 2a:** Statistical results of VISIA red region feature count.

Red region feature count	Control group	Experimental Group I	Experimental Group II	Experimental Group III	*F*	*p*
Before treatment	78.67 ± 8.27	74.13 ± 5.34	76.87 ± 4.00	75.40 ± 4.24	1.745	0.168
The first treatment	74.67 ± 6.75	64.53 ± 4.81	66.73 ± 5.24	43.60 ± 9.56	56.226	**< 0.001**
The second treatment	69.60 ± 6.24	55.67 ± 5.43	54.47 ± 3.27	33.47 ± 6.77	106.538	**< 0.001**
The third treatment	64.40 ± 6.32	47.73 ± 6.08	40.27 ± 6.70	25.07 ± 7.14	92.851	**< 0.001**

*Note:* One‐way ANOVA was used to analyze the red region feature count data detected by the VISIA skin detector in four groups at four time nodes. The results passed the homogeneity test of variance, and one‐way ANOVA could be used. There was no significant difference in the red zone characteristic count among the four groups before treatment, but there was a significant difference among the four groups after the first, second, and third treatment (*p*‐value < 0.05). Statistically significant results (*p*‐value < 0.05) are shown in bold, and results are expressed as the mean ± standard deviation.

**TABLE 2b:** The least significant difference (LSD) method was used to compare the red region feature count among the four groups.

Red region feature count	12	13	14	23	24	34
The first treatment	**< 0.001**	**0.002**	**< 0.001**	0.383	**< 0.001**	**< 0.001**
The second treatment	**< 0.001**	**< 0.001**	**< 0.001**	0.559	**< 0.001**	**< 0.001**
The third treatment	**< 0.001**	**< 0.001**	**< 0.001**	**0.003**	**< 0.001**	**< 0.001**

*Note:* The results of LSD pairwise comparison showed that the characteristic count of the red region in the three time nodes of the experiment three groups was significantly lower than that in the control group; Experimental Group III was significantly lower than the other three groups; after the first and second treatment, there was no statistical difference between Experimental Group I and Experimental Group II; and after the third treatment, there was a significant difference between Experimental Group I and Experimental Group II. Statistically significant results (*p*‐value < 0.05) are shown in bold.

### 3.2 Patient Self‐Assessment

**FIGURE 2:**
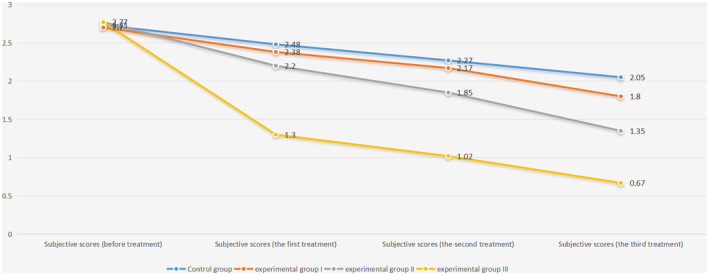
Trend plot of patient subjective ratings.

Subjective scores, reflecting patients' self‐assessment of their symptoms at multiple time points (Table 3a; Figure 2; Figure S2), were analyzed using one‐way ANOVA, preceded by tests for homogeneity of variance. No significant differences were identified at the pretreatment stage. However, postintervention, significant improvements were noted after each treatment session. Notably, the third group consistently recorded the lowest scores, suggesting the most favorable therapeutic outcomes. The differences between Group I and the control group, as well as between Group I and Group II, reached significance after the second and third treatments, respectively, indicating a progressive enhancement in patients' self‐perceived skin condition (Table 3b).

**TABLE 3a:** Statistical results of patients' subjective scores.

Subjective scores	Control group	Experimental Group I	Experimental Group II	Experimental Group III	*F*	*p*
Before treatment	2.73 ± 0.37	2.70 ± 0.34	2.77 ± 0.20	2.77 ± 0.22	0.177	0.911
The first treatment	2.48 ± 0.36	2.38 ± 0.31	2.20 ± 0.24	1.30 ± 0.33	44.917	**< 0.001**
The second treatment	2.27 ± 0.26	2.17 ± 0.32	1.85 ± 0.25	1.02 ± 0.20	71.167	**< 0.001**
The third treatment	2.05 ± 0.19	1.80 ± 0.30	1.35 ± 0.25	0.67 ± 0.26	85.746	**< 0.001**

*Note:* The subjective scores of perceived symptoms related to skin barrier damage in four groups at four time nodes were analyzed by one‐way ANOVA. The results were tested for homogeneity of variance, and one‐way ANOVA could be used. There was no significant difference in subjective scores among the four groups before treatment, and there was a significant difference in subjective scores among the four groups after the first, second, and third treatment (*p*‐value < 0.05). Statistically significant results (*p*‐value < 0.05) are shown in bold, and results are expressed as the mean ± standard deviation.

**TABLE 3b:** The least significant difference (LSD) method was used to compare the subjective scores among the four groups.

Subjective scores	12	13	14	23	24	34
The first treatment	0.384	**0.016**	**< 0.001**	0.114	**< 0.001**	**< 0.001**
The second treatment	0.298	**< 0.001**	**< 0.001**	**0.002**	**< 0.001**	**< 0.001**
The third treatment	**0.009**	**< 0.001**	**< 0.001**	**< 0.001**	**< 0.001**	**< 0.001**

*Note:* The LSD pairwise comparison showed that the subjective scores of patients in Experimental Group III were significantly lower than that of the other three groups. After the first and second treatments, there was no statistical difference in the subjective scores between the Experimental Group I and the control group, and there was no significant difference in the subjective scores until the third treatment. After the first treatment, there was no statistical difference between the subjective scores of Experimental Group I and Experimental Group II, and there was no significant difference until the second treatment. Statistically significant results (*p*‐value < 0.05) are shown in bold.

### 3.3 Objective Clinical Physician Scores

**FIGURE 3:**
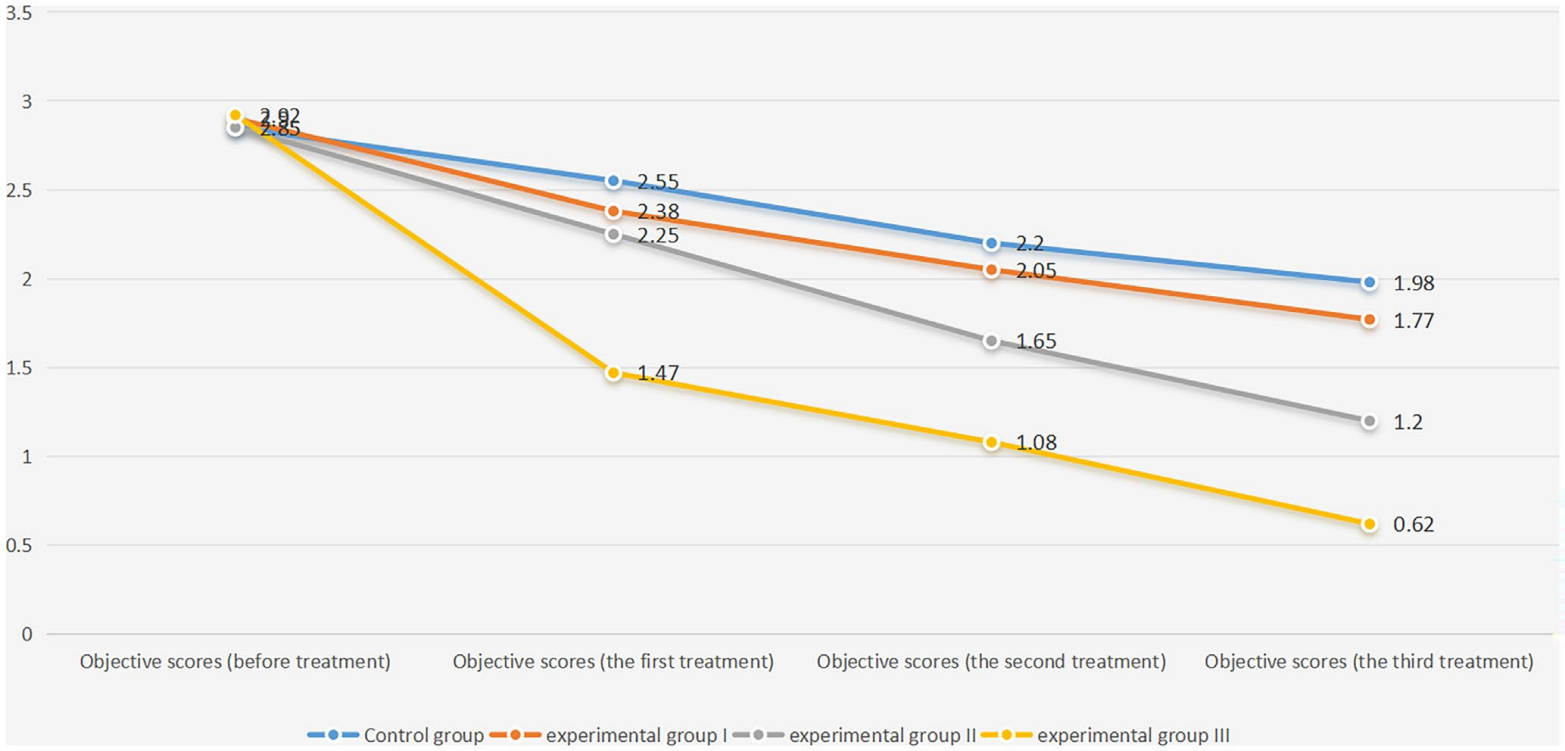
Trend plot of objective ratings by physicians.

The objective clinical assessments, as indicated by physician scores (Table 4a; Figure 3; Figure S3), were initially homogeneous, with no significant differences observed following one‐way ANOVA. However, after the initial three treatments, statistical differences emerged, with Group III consistently demonstrating the lowest scores using the LSD test, signifying the most effective therapeutic response. Not until the second and third treatments did differences between Group I and the control group, as well as between Group I and Group II, become evident (Table 4b), underscoring the treatment's progressive effect.

**TABLE 4a:** Statistical results of objective scoring by physicians.

Objective scores	Control group	Experimental Group I	Experimental Group II	Experimental Group III	*F*	*p*
Before treatment	2.85 ± 0.18	2.90 ± 0.18	2.85 ± 0.21	2.92 ± 0.15	0.527	0.666
The first treatment	2.55 ± 0.14	2.38 ± 0.27	2.25 ± 0.23	1.47 ± 0.36	50.021	**< 0.001**
The second treatment	2.20 ± 0.25	2.05 ± 0.24	1.65 ± 0.28	1.08 ± 0.32	49.400	**< 0.001**
The third treatment	1.98 ± 0.27	1.77 ± 0.24	1.20 ± 0.32	0.62 ± 0.34	64.791	**< 0.001**

*Note:* One‐way ANOVA was used in four groups at four time nodes for blind evaluation and objective scores by doctors based on patient photos and VISIA skin test results. The results passed the homogeneity test of variance, and one‐way ANOVA could be used. There was no significant difference in the objective scores among the four groups before treatment, and there was a significant difference among the four groups after the first, second, and third treatment (*p*‐value < 0.05). Statistically significant results (*p*‐value < 0.05) are shown in bold, and results are expressed as the mean ± standard deviation.

**TABLE 4b:** The least significant difference (LSD) method was used to compare the objective scores among the four groups.

Objective scores	12	13	14	23	24	34
The first treatment	0.088	**0.003**	**< 0.001**	0.170	**< 0.001**	**< 0.001**
The second treatment	0.141	**< 0.001**	**< 0.001**	**< 0.001**	**< 0.001**	**< 0.001**
The third treatment	**0.049**	**< 0.001**	**< 0.001**	**< 0.001**	**< 0.001**	**< 0.001**

*Note:* LSD pairwise comparison showed that patients in Experimental Group III were significantly lower than those in the other three groups. After the first and second treatments, there was no statistical difference between the objective scores of doctors in Experimental Group I and the control group, and there was no significant difference in the objective scores until the third treatment. After the first treatment, there was no statistical difference between the objective scores of Experimental Group I and Experimental Group II, and there was no significant difference until the second treatment. Statistically significant results (*p*‐value < 0.05) are shown in bold.

### 3.4 Repeated Measures ANOVA

A repeated measures ANOVA on the three assessment indicators showed a positive correlation between treatment frequency and skin barrier repair outcomes. This suggests that each session led to an improvement in skin barrier status relative to the previous state (Tables S6–S8), highlighting the cumulative benefits of the treatment.

### 3.5 VISIA Red Area Images and Clinical Photographs

**FIGURE 4:**
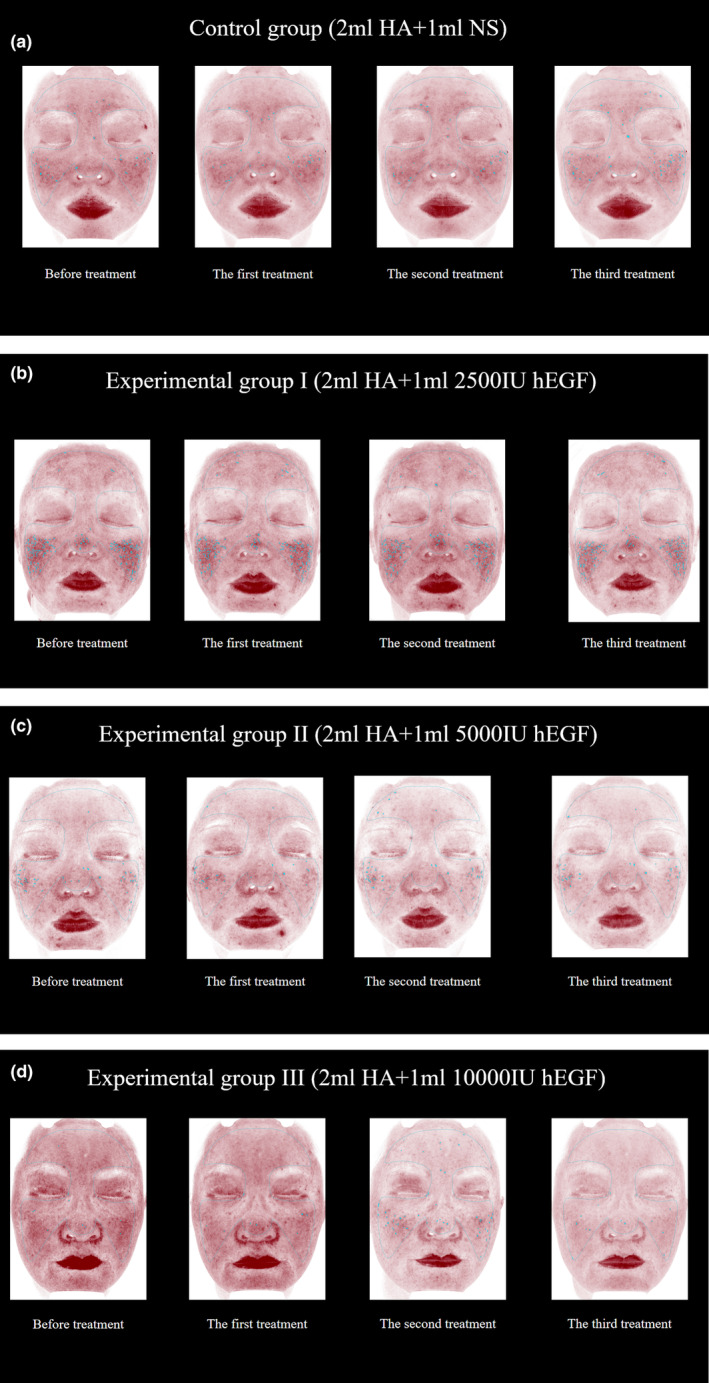
The VISIA red area compared before and after treatment: (a) control group, (b) Experimental Group I, (c) Experimental Group II and (d) Experimental Group III.

**FIGURE 5:**
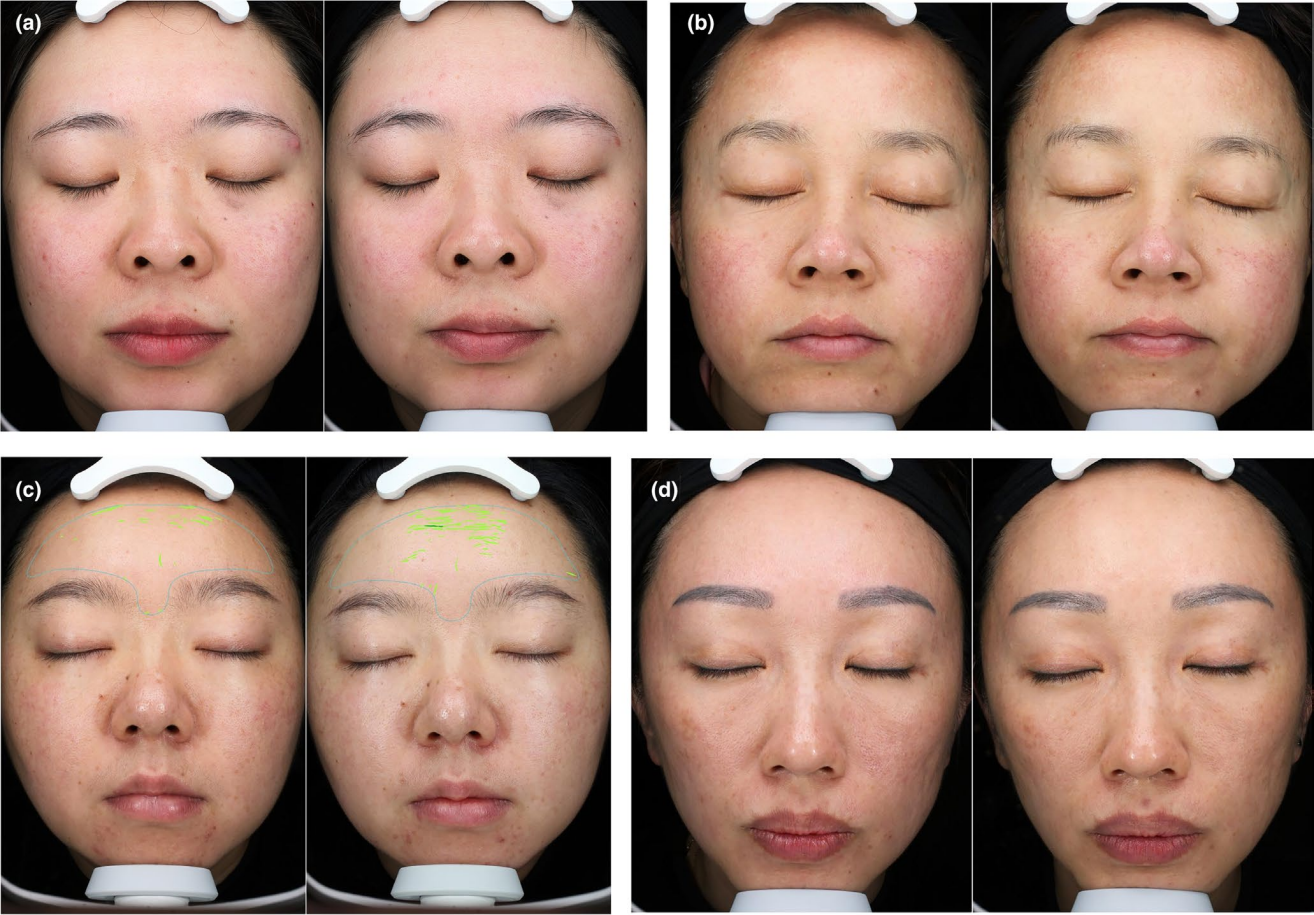
The clinical photographs compared before and after treatment: (a) control group, (b) Experimental Group I, (c) Experimental Group II and (d) Experimental Group III.

Pre‐ and posttreatment VISIA red area images (Figure 4a–d) and clinical photographs (Figure 5a–d) for all groups visually corroborate these improvements. The nonsignificance of the three assessment indicators prior to treatment confirms the successful randomization of the experimental groups and the study design's validity.

### 3.6 Dose‐Dependent Efficacy of hEGF

Group III, administered 10 000 IU hEGF, exhibited a notably higher rate of skin barrier repair compared to other groups, illustrating the dose‐dependent nature of hEGF's efficacy in skin repair.

### 3.7 Overall Effectiveness and Safety

The average posttreatment assessments across all experimental groups, including VISIA red area feature counts, patient self‐assessment scores, and physician objective scores, were consistently higher than pretreatment values. This indicates the overall effectiveness of the treatment protocol. The total improvement rates in the red zone after three treatments were as follows: 18.01% for the control group, 35.63% for Group I, 47.32% for Group II, and 66.80% for Group III, demonstrating a correlation between hEGF concentration and skin barrier recovery. Importantly, with 60 participants, no posttreatment complications were reported, affirming the safety of the treatment protocol.

## 4 Discussion

Our study tackles the prevalent issue of skin barrier damage in the high‐altitude region of Yunnan Province, China, by demonstrating the therapeutic benefits of combining non‐crosslinked HA with hEGF for skin repair. The cumulative effect of multiple treatments significantly enhanced the therapeutic outcome, suggesting a dose‐dependent response.

Intradermal HA injections are known to enhance skin properties such as smoothness, hydration, and elasticity, and promote collagen production [12]. However, in the high‐UV environment of the Yunnan Plateau, HA alone was insufficient for effective skin barrier repair due to the profound damage to the epidermal barrier structure. The inability of HA to inhibit transepidermal water loss results in limited and transient therapeutic effects.

The synergistic use of HA and hEGF, which form a biomembrane, has been reported to prolong the residence time of hEGF at the wound site, thereby increasing its stability and accelerating the healing process [10]. hEGF is recognized for its role in skin cell regeneration and proliferation, where it stimulates collagen and HA synthesis, enhancing skin elasticity and hydration [13]. Additionally, hEGF promotes keratinocyte proliferation and re‐epithelialization, which are critical for the rapid repair of the stratum corneum [14, 15]. The topical application of hEGF ensures a high concentration at the wound site, accelerating these biological effects and effectively reducing the time required for skin barrier repair [16]. However, the molecular size of hEGF, which exceeds 15 000 Da, can limit its penetration through the stratum corneum. Microneedling provides a pathway for penetration by puncturing the skin at a controlled depth, inducing an intrinsic wound healing response that promotes collagen formation and neovascularization, essential for repairing the skin barrier damaged by UV radiation [17, 18, 19, 20]. In our study, a single‐use skin roller was utilized to create physical channels, enabling hEGF to effectively target keratinocytes, accelerate proliferation, and induce normal differentiation, thereby restoring the barrier function of the stratum corneum [21, 22, 23]. The experimental groups treated with 2500 and 5000 IU hEGF required a cumulative number of treatments to show significant differences, likely due to the short half‐life of hEGF and its susceptibility to degradation in a damaged environment [24]. The most efficient method for skin barrier repair identified in this study was the combination of non‐crosslinked HA injection with microneedling and full‐face application of 10 000 IU hEGF, which was administered without any reported local complications such as induration or erythema. It is noted that both too low and too high concentrations of hEGF can reduce the reparative effect on the epidermal layer; low concentrations due to degradation, and high concentrations due to excessive collagen expression leading to fibrosis and scarring. An hEGF concentration of 10 μg/g was found to be optimal for promoting wound healing, aligning with the saturation point of epidermal growth factor receptors (EGFRs) on the cell surface, beyond which no further healing promotion occurs [25]. This underscores the importance of an optimal hEGF concentration in treatment protocols for effective skin barrier repair.

In this study, we introduce a novel approach to skin barrier repair by combining non‐crosslinked sodium HA injection with post‐microneedle hEGF treatment, targeting the restoration of keratinocyte structure and epidermal hydration. This method has demonstrated greater efficacy in addressing the prevalent skin challenges faced by inhabitants of the Yunnan Plateau region. However, the study acknowledges certain limitations. The conservative microneedle depth of 0.5 mm might have restricted the full efficacy of the epidermal growth factor, potentially due to inadequate penetration. A more extensive range of hEGF concentrations could have been explored to determine the optimal dosage for skin barrier repair. Additionally, the 4‐month treatment duration and variable adherence to sun exposure restrictions among participants, along with variations in geographic living conditions and skincare habits, could have influenced the outcomes. Furthermore, the study design could be enhanced by incorporating a half‐face control experiment, applying hEGF to the full face for more robust validation of the treatment effects. What is more, while we acknowledge the value of having clinical photos from all treatment sessions, we only collected clinical photos before treatment and after the third treatment session due to logistical constraints and the focus on the final therapeutic outcomes. Clinical photos of all stages should be included to further enhance the persuasive conclusion in the future. Lastly, the exploration of new treatment modalities and an increased subject sample size would also strengthen the findings and generalizability of the study.

## 5 Conclusion

The combination of hEGF and non‐crosslinked HA has shown a more effective capability in repairing skin barrier damage in high‐altitude areas. There exists a positive correlation between the concentration of hEGF and the efficacy of skin barrier repair. To obtain the most optimal results in skin barrier restoration, a regular and scientific treatment regime must be supplemented by good daily skincare routines for the patients. This integrated method guarantees that the skins natural defenses are strengthened and that the healing process is supported both clinically and through personal care.

## Author Contributions

Ao He and Boyan Liu contributed equally to this work as first authors. They were responsible for the study design, data collection, analysis, and interpretation. They also drafted the manuscript and approved the final version for submission. Yingjian Hua was involved in clinical data collection and patient recruitment. He provided valuable insights into the dermatological assessments and contributed to the discussion section of the manuscript. Zhuo Gong contributed to the statistical analysis of the study data. He ensured the accuracy and reliability of the results and helped in interpreting the findings in the context of the study objectives. Fengshan Gan was responsible for overseeing the clinical procedures and ensuring the ethical conduct of the study. He also participated in the review and revision of the manuscript, providing critical feedback on the clinical aspects. Qingzhu Zhou contributed to the conceptualization of the study and the development of the treatment protocol. She was instrumental in securing funding for the project and provided guidance throughout the research process. Songmei Wang is a corresponding author who contributed significantly to the study. She was involved in the overall supervision of the project, including the design, execution, and analysis of the study. She also played a pivotal role in the preparation of the manuscript and the decision to submit the work for publication. Xian Zhao is the other corresponding author who shares responsibility for the study. She was responsible for the overall coordination and management of the research team. She also contributed to the critical revision of the manuscript for important intellectual content and provided final approval of the version to be published.

## Ethics Statement

This study was conducted in accordance with the ethical standards as prescribed by the International Council for Harmonization of Technical Requirements for Pharmaceuticals for Human Use (ICH) and the Chinese Good Clinical Practice (GCP). The research was approved by the Ethics Committee of an affiliated hospital on [2023‐5‐12], with the approval number YLS2023‐17. The study was granted ethical clearance following a thorough review process, ensuring the protection of the rights, safety, and well‐being of all human subjects involved. Informed consent was obtained from each participant prior to their inclusion in the study. The consent procedure was explained in detail, and participants were assured of confidentiality and the voluntary nature of their participation.

## Conflicts of Interest

The authors declare no conflicts of interest.

## Supporting information


Tables S1–S8.

Figure S1.

Figure S2.

Figure S3.

## Data Availability

The data that supports the findings of this study are available in the Supporting Information of this article.
