# Supplementary figures and images for: Clinical Study of Intradermal Injection of Non‐Crosslinked Sodium Hyaluronate Combined With Human Epidermal Growth Factor in the Treatment of Skin Barrier Injury in Plateau Area
Source: J Cosmet Dermatol. 2024 Dec 24;24(2):e16727. doi: 10.1111/jocd.16727 (PMC11837232; doi:10.1111/jocd.16727)

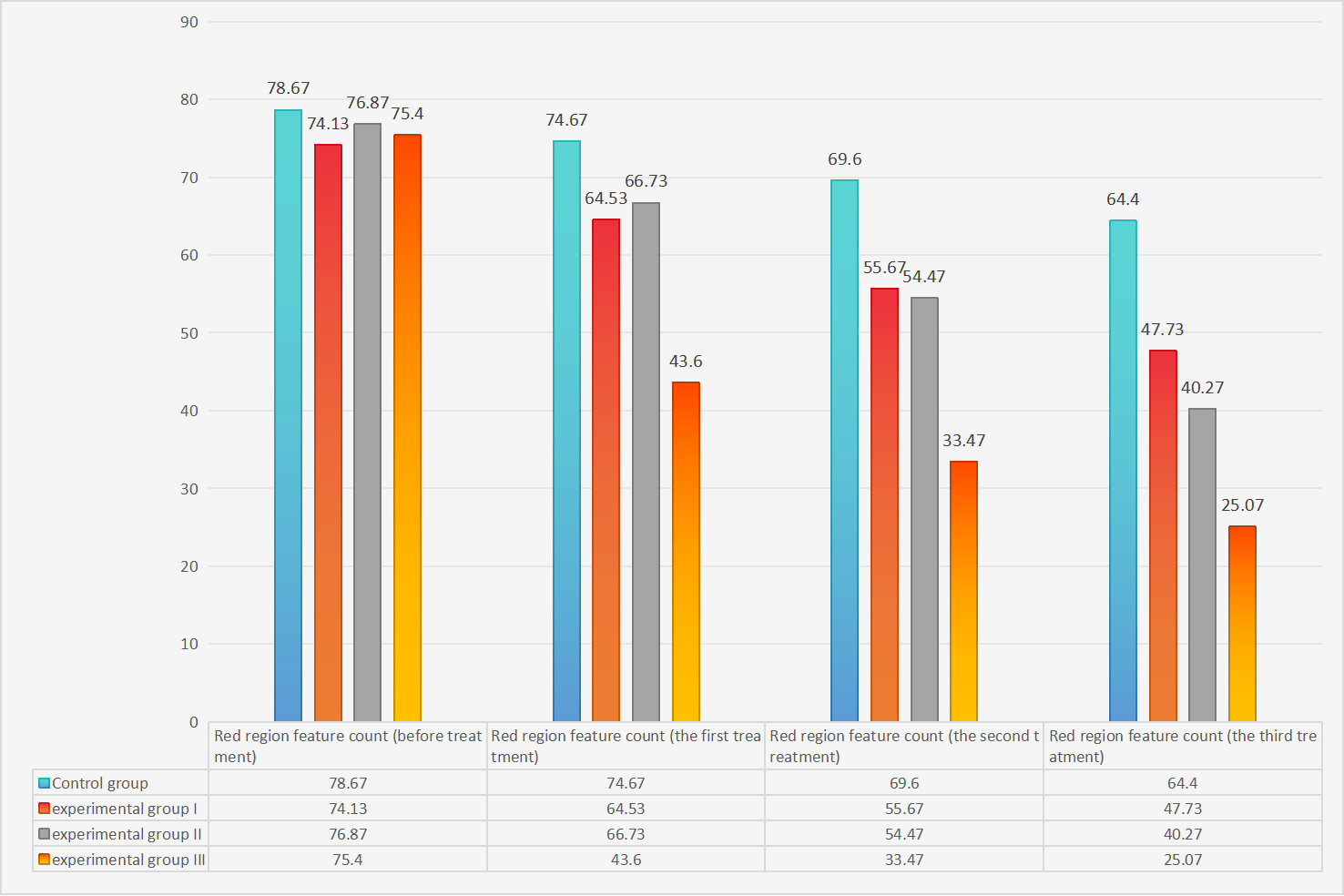

Supplement: Supplementary file 2 — Figure S1. [file JOCD-24-e16727-s001.tif]

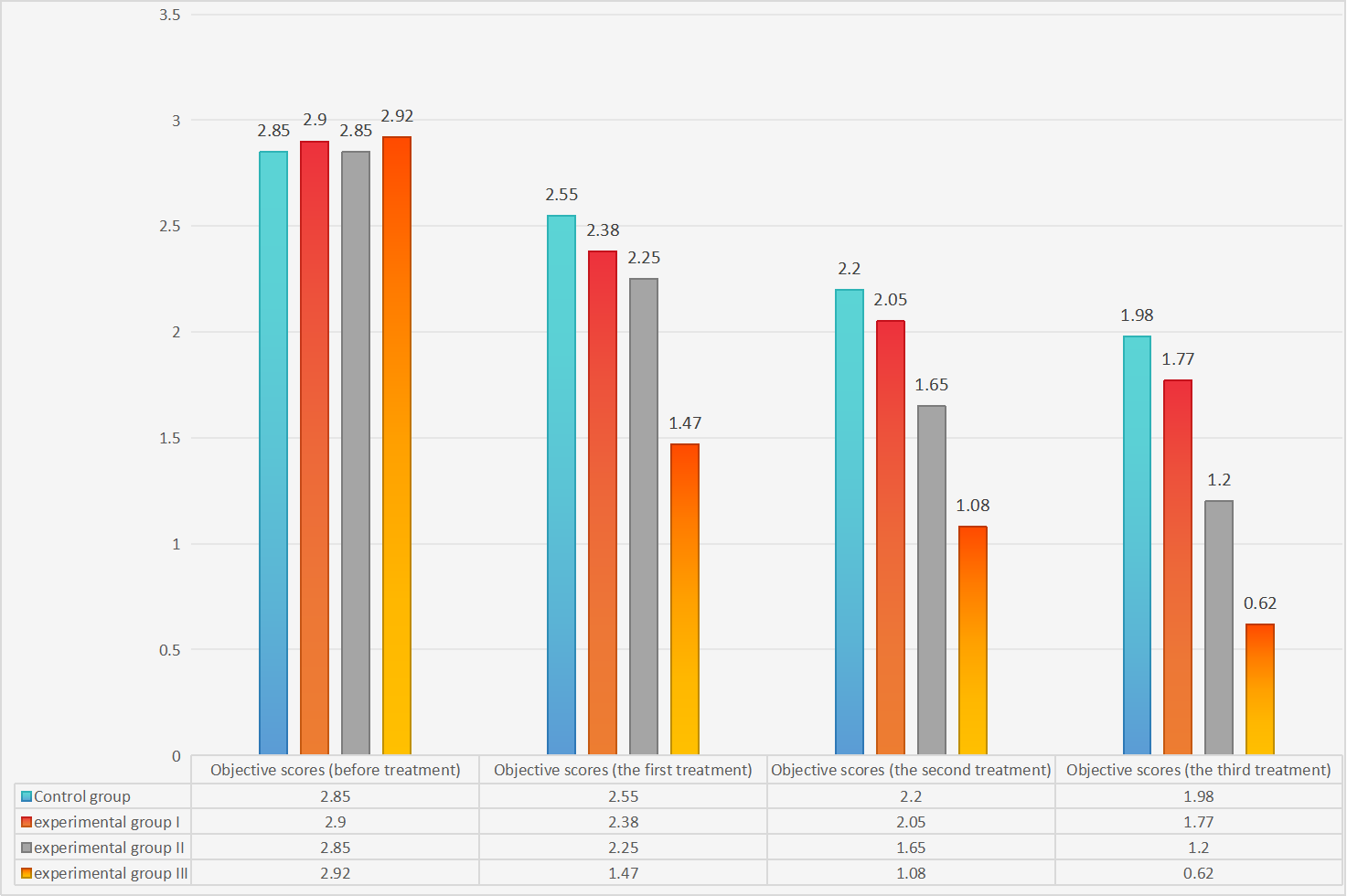

Supplement: Supplementary file 3 — Figure S2. [file JOCD-24-e16727-s002.tif]

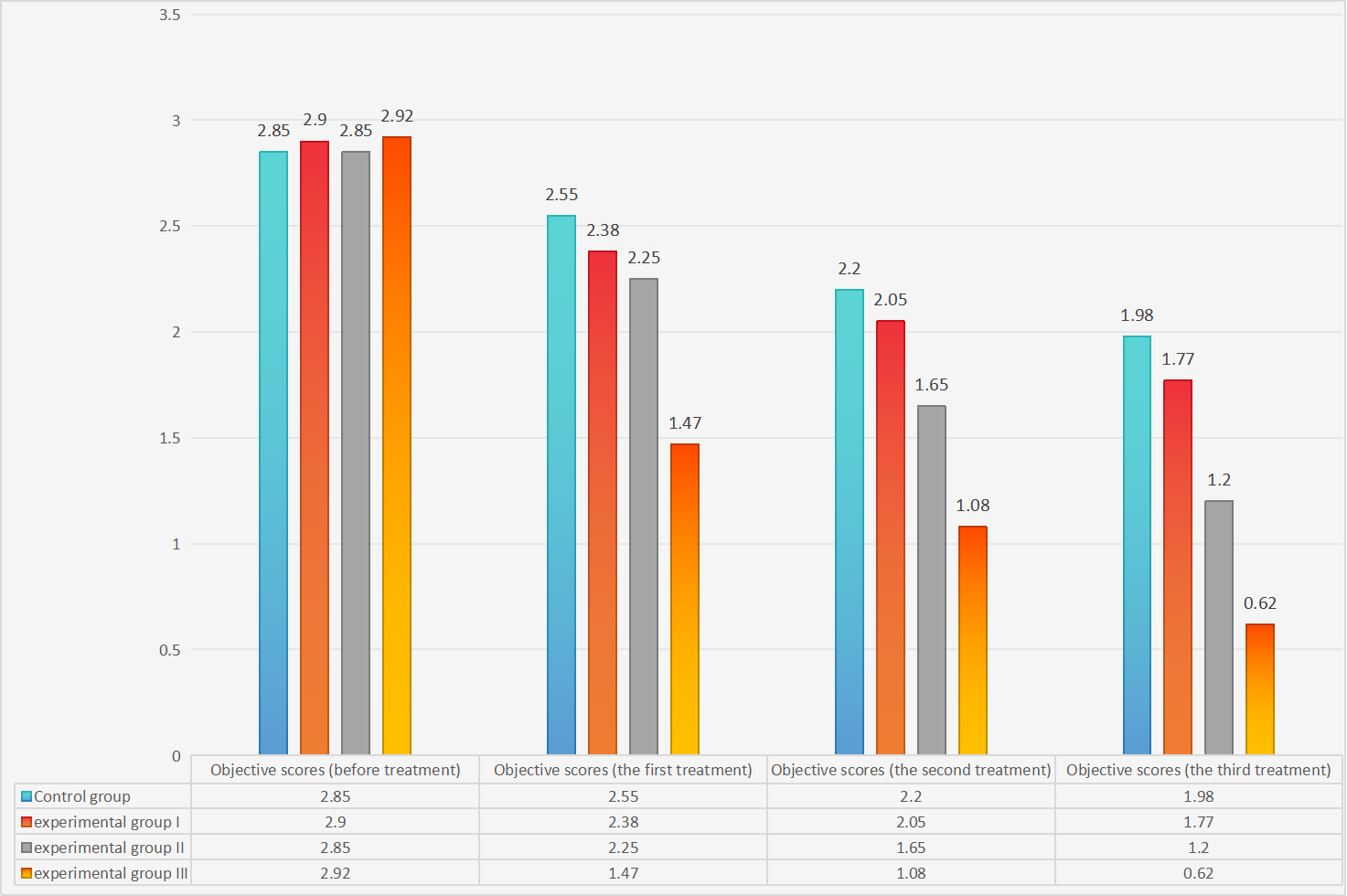

Supplement: Supplementary file 4 — Figure S3. [file JOCD-24-e16727-s004.tif]
